# Innervation of the distal part of the vastus medialis muscle is endangered by splitting its muscle fibers during total knee replacement: an anatomical study using modified Sihler’s technique

**DOI:** 10.1080/17453674.2020.1851459

**Published:** 2020-11-24

**Authors:** Bettina Pretterklieber, Alfred Ungersböck, Michael L Pretterklieber

**Affiliations:** a Division of Anatomy, Center for Anatomy and Cell Biology, Medical University of Vienna, Vienna;; b Department of Orthopaedic and Trauma Surgery, Federal Hospital Neunkirchen, Neunkirchen, Austria

## Abstract

Background and purpose — The distal part of the vastus medialis muscle is an important stabilizer for the patella. Thus, knowledge of the intramuscular nerve course and branching pattern is important to estimate whether the muscle’s innervation is at risk if splitting the muscle. We determined the intramuscular course of the nerve branches supplying the distal part of the vastus medialis muscle to identify the surgical approach that best preserves its innervation.

Material and methods — 8 vastus medialis muscles from embalmed anatomic specimens underwent Sihler’s procedure to make soft tissue translucent while staining the nerves to study their intramuscular course. After dissection under transillumination using magnification glasses all nerve branches were evaluated.

Results — The terminal nerve branches were located in different layers of the muscle and ran mostly parallel but also transverse to the muscle fibers. In half of the cases, the latter formed 1 to 3 anastomoses and coursed close to the myotendinous junction. Additionally, most of the branches extended into the ventromedial part of the knee joint capsule.

Interpretation — To preserve the innervation of the distal part of the vastus medialis muscle, any split of the muscle during surgical approaches to the knee joint should be avoided.

Based on the different orientation of the vastus medialis muscle fiber bundles, Lieb and Perry (1968) were the first authors who described 2 parts, i.e., the vastus medialis longus and the vastus medialis obliquus. Whether these 2 parts really exist as 2 individual muscles is debated (Speakman and Weisberg 1977, Hubbard et al. 1997, Peeler et al. 2005, Smith et al. 2009). In any event, these caudal oblique coursing muscle fiber bundles, which insert into the cranial part of the medial margin of the patella, are said to be essential for so-called patellar tracking (Goh et al. 1995, Toumi et al. 2007, Lin et al. 2008).

The vastus medialis muscle is innervated by the femoral nerve. The branch for the distal portion of the vastus medialis muscle courses outside the adductor canal before it enters the muscle in the middle third of its belly. Some final sensory twigs of this branch have been reported to reach also the ventromedial part of the capsule of the knee joint, thus apparently being part of its proprioception (Thiranagama 1990, Horner and Dellon 1994, Nozic et al. 1997). Both these functions of these nerves seem to play an important role for the function of the knee joint. Therefore, these nerves and their terminal branches should be preserved as much as possible during medial approaches to the knee joint.

Surgical incision into the knee joint for total knee replacement can be done in different ways: the classical medial parapatellar approach (Cooper et al. 1999), the subvastus approach (Erkes 1929, Halder et al. 2009), and as a compromise the midvastus approach. Here, the incision divides the distal part of the vastus medialis (Hube et al. 2009), which may interrupt its innervation. Parentis et al. (1999) and Kelly et al. (2006) have already reported abnormal electromyographical findings following this approach.

We determined the intramuscular course and the final destination of the nerve branches within the distal part of the vastus medialis muscle to identify the approach that best preserves its innervation.

## Material and methods

We examined 8 vastus medialis muscles together with the adjacent part of the capsule of the knee joint. They were taken from the right leg of 3 male and 5 female embalmed anatomic specimens from our student dissection course. The age of the deceased individuals was on average 80 years (56–94). The bodies of the deceased persons were perfused and immerged with a low-percentage formalin-phenol solution. We used muscles only from sites without any signs of surgical intervention within the anterior femoral region and knee joint.

Immediately after excising the muscles, we performed whole-mount nerve staining using the modified Sihler’s technique (Liu et al. 1997, Mu and Sanders 2010) to render soft tissue translucent or transparent while staining the nerves to study their intramuscular course and pattern. As the muscles originated from formalin-fixed specimens, we skipped the first step, i.e., fixation in 10% formalin. This modification has already successfully been done by Sekiya et al. (2005). To make the tissue transparent we macerated the muscles with a 3% potassium hydroxide (KOH) solution with 0.2 mL 3% hydrogen peroxide per 100 mL. The next step was decalcification in Sihler’s solution I (1 equivalent glacial acetic acid, 1 equivalent glycerin, 6 equivalents 1% aqueous chloral hydrate). After that, we stained the tissues using Sihler’s solution II (1 equivalent stock Ehrlich’s hematoxylin, 1 equivalent glycerin, 6 equivalents 1% aqueous chloral hydrate). To decolor the muscle fibers and connective tissue again, we destained the muscles again using Sihler’s solution I. Following neutralization in a 0.05% lithium carbonate solution, we put the muscles into 50% aqueous glycerin for clearing. Finally, we stored them in 100% glycerin with a few thymol crystals as antiseptic agent.

**Figure 1.**
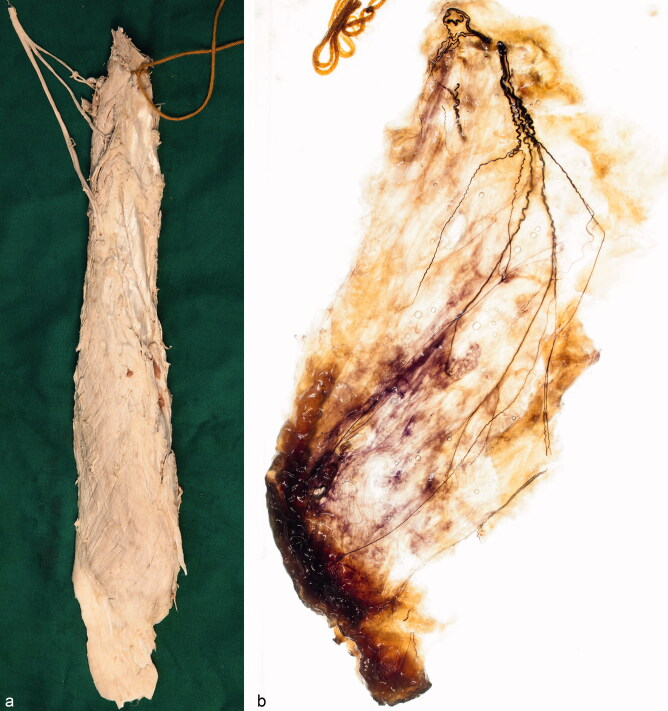
Right vastus medialis muscle of a 56-year-old woman. The muscle was removed from a formalin-fixed specimen and marked with yellow yarn (a). The same muscle after Sihler’s procedure transilluminated by a white light transilluminator. The nerve branches are stained dark blue whereas the muscle fibers show a transparent lavender color after the destaining and clearing process. Some of the superficial muscle fibers were removed to show the whole course of all nerve branches. The ventrolateral part of the capsule of the knee joint appears amber (b).

To record the course of the fine nerve twigs, which innervate the distal part of the vastus medialis muscle and the adjacent part of the capsule of the knee joint, we dissected the nerves under transillumination with a white light transilluminator using magnification glasses. We removed some of the superficial muscle fibers to make the whole muscle more transparent. To gain a better view of the course of the nerve branches, intramuscular arteries and veins were mostly resected. To compare the 2 medial minimal invasive approaches under discussion, we simulated them on 2 non-embalmed anatomic specimens. We took photographs with a digital reflex camera. Figure 1 shows an example of a vastus medialis muscle before and after Sihler’s procedure and further dissection.

### Ethics and funding

The bodies had been donated to medical education and research at our university. In addition to the informed consent of the body donors, approval was obtained from the ethics committee of our university (approval number: 1826/2017).No funding was received for this study. 

## Results

**Figure 2.**
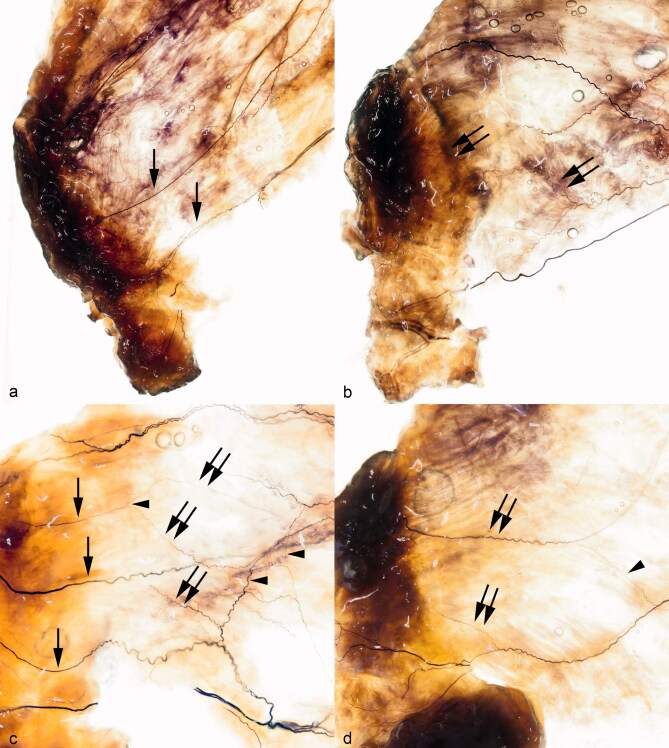
Detailed view of the transilluminated distal part of 4 right vastus medialis muscles and the adjacent ventromedial part of the capsule of the knee joint: (a) from a 56-year-old woman, (b) from an 83-year-old woman, (c) from a 67-year-old woman, (d) from a 82-year-old man. The nerve branches are located in different layers of the muscle. They run parallel (single arrows) or transverse (double arrows) to the muscle fibers and finally reach the ventromedial part of the capsule of the knee joint. In half of the subjects, they build 1 or more anastomoses (arrowhead) within the distal part of the vastus medialis muscle.

In all 8 vastus medialis muscles, 4 to 8 nerve branches were recorded within the distal part of this muscle. Consistently, the most distal branch ran alongside the posterior margin of the muscle and gave rise to several other branches. All branches were located in different layers of the muscle. Although they coursed more or less parallel to the muscle fibers (Figure 2a), in 7 out of 8 cases, 1 to 7 of the branches also crossed the muscle fibers in a transverse direction (Figure 2b–d). These traversing branches often coursed close to the myotendinous junction. Due to further division, 4 to 9 branches extended into the ventromedial part of the fibrous capsule of the knee joint. In 4 cases, 1 to 3 anastomoses between 2 of the nerve branches were observed running perpendicular to the muscle fibers (Figure 2c–d). These anastomotic branches also frequently coursed close to the myotendinous junction. In addition to this primary pattern, the nerve branches often formed networks (Figure 2c). 

## Discussion

Our study is the first to describe the intramuscular course of the nerve branches innervating the vastus medialis muscle using the modified Sihler’s technique. This technique has been reported to be superior to microdissection, or 3D reconstruction for observing motor nerve supply patterns in muscles, as the 3D structure of the whole specimen can be preserved (Mu and Sanders 2010). The modified Sihler’s technique is a time-consuming process, which requires several weeks or months (Mu and Sanders 2010). Thus, studies using this method are usually based on a small sample size, e.g., 3 human tongues (Doty et al. 2009), abdominal walls from 5 rats (Calguner et al. 2006), or the posterior cricoarytenoid muscle of 10 dogs (Drake et al. 1993). We used 8 specimens. Due to the use of this method, it was possible to trace the nerves without severing the topographical relationship to each other and to the muscle fibers. It revealed that the terminal nerve branches within all layers of the distal part of the vastus medialis muscle course parallel to the muscle fibers but also traverse them. Thereby they build anastomoses with each other. These traversing branches course close to the myotendinous junction. The results are a valuable addition to the findings of Ehler et al. (1959) and Jojima et al. (2004), who described the course of the nerves only by pure macroscopic dissection, preventing them from analyzing the branches in the detailed way we did by using Sihler’s procedure, i.e., the small anastomoses and traversing branches. As the innervation patterns of each vastus medialis muscle in detail seems to be prone to interindividual variation; one cannot predict if an intramuscular nerve branch will be severed by cutting through or between the muscle fibers. The anastomoses and the transverse coursing branches seem thereby especially vulnerable.

**Figure 3.**
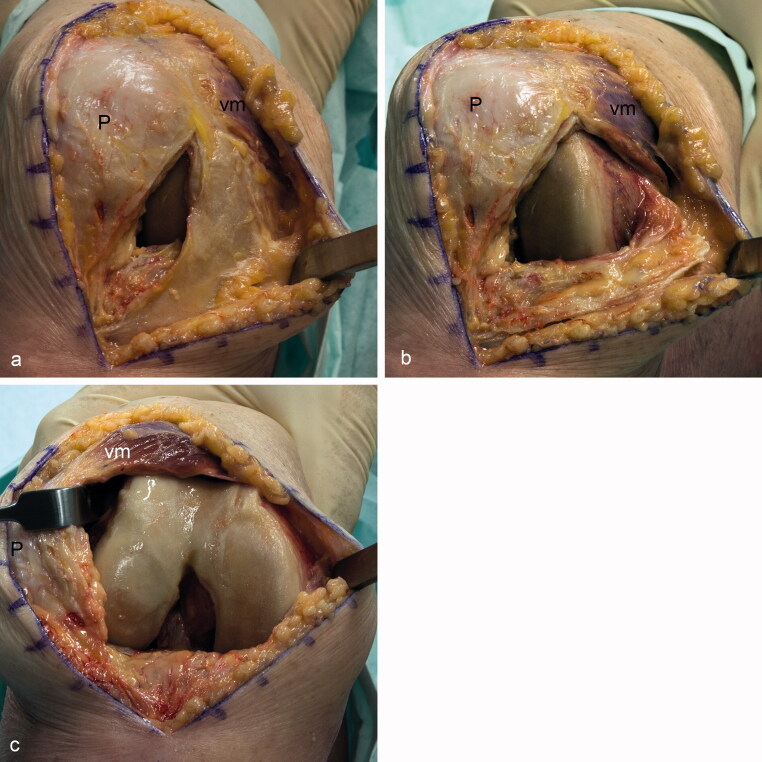
Simulation of the subvastus approach performed in a non-embalmed anatomic specimen. The vastus medialis muscle (vm) is preserved during the subvastus approach, as the dissection follows its caudal border (a, b). To gain more space, the vastus medialis muscle can be mobilized from the tendon of the adductor magnus by blunt dissection. The muscle together with the patella (P) can then be lateralized to get access to the joint surfaces (c).

**Figure 4.**
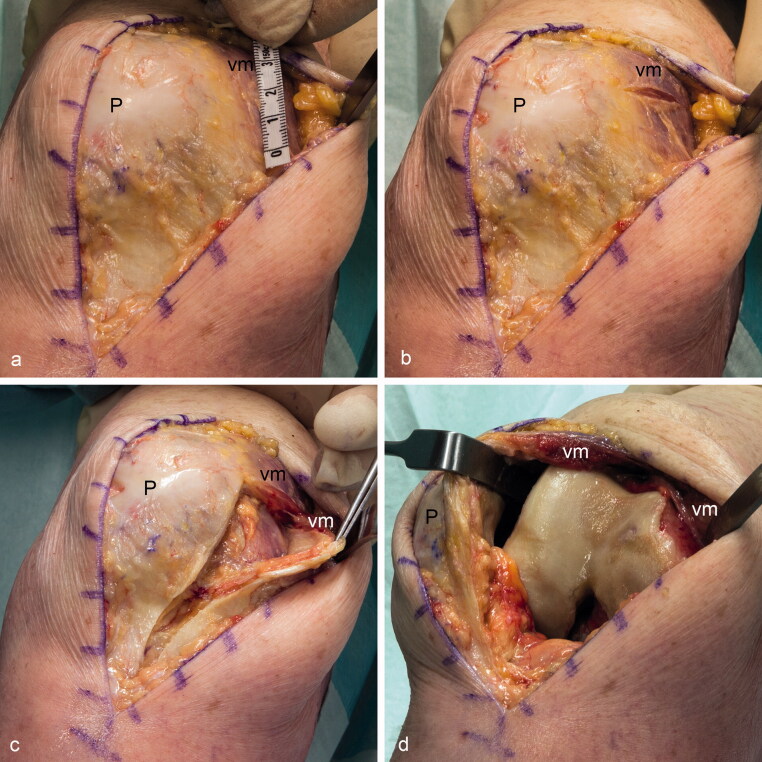
Simulation of the midvastus approach performed in a non-embalmed anatomic specimen. The distal part of the vastus medialis muscle (vm) is split about 3 cm cranial from its caudal border (a, b, c). The cranial part of the muscle together with the patella (P) can be lateralized to gain access to the joint (d).

There are different medial approaches into the knee joint for total knee replacement. Each has its advantages and disadvantages. During the standard medial parapatellar approach, the tendon of the vastus medialis muscle is interrupted. This approach leads to a good overview of the joint and can be done in nearly all patients. However, using this procedure, the extensor apparatus of the knee joint is severed, which was seen in former times as a reason for postoperative patellar tracking problems (Clayton and Thirupathi 1982, Cooper et al. 1999). Therefore, a lateral release is sometimes performed simultaneously (Keblish 2002). In addition, nowadays these problems are supposed to be created, rather, by a malrotation of the implant (van Rensch et al. 2020). The subvastus approach (Figure 3) preserves the integrity of the vastus medialis muscle (Erkes 1929). Hofmann et al. (1991) reported an equivalent exposure compared with the parapatellar approach. However, some authors are convinced that this procedure is more difficult to perform and only provides diminished visibility of the joint surfaces (Keblish 2002, Halder et al. 2009). As a compromise, the midvastus approach (Figure 4) has been developed. The cutting line is similar to the medial parapatellar approach leaving the vastus medialis muscle in continuation with its aponeurosis but splitting the distal part of the muscle parallel to its fibers (Dalury and Jiranek 1999). This approach is also said to be more difficult than the parapatellar approach, due to the lesser reachability of the joint surfaces (Keblish 2002, Hube et al. 2009). Cooper et al. (1999) stated that this approach does not harm the innervation of the distal part of the vastus medialis muscle. They have postulated that one can cut 4 cm through the muscle and an additional 2 cm remain as a safe distance for blunt dissection. Apparently, the authors have only regarded the extramuscular course of the nerves supplying the distal part of the vastus medialis muscle. In contrast, our results suggest that the traversing branches can almost reach the myotendinous junction, indicating the safe zone of 4 cm stated by Cooper et al. (1999) to be inappropriate. Indeed, the midvastus approach is controversial. Parentis et al. (1999) reported abnormal postoperative electromyographical recordings in 9 of 21 knees undergoing the midvastus approach. In 2 of these 9 knees, these irregularities still exist after more than 5 years, even though without discernible functional deficits (Kelly et al. 2006). In one-third of the midvastus group observed by Jojima et al. (2004) main nerve branches within the distal part of the vastus medialis had been disrupted. On the other side, Dalury et al. (2008) found similar postoperative electromyographical recordings following the medial parapatellar and the midvastus approaches. As they only cut sharp for a distance of 5 cm, they were convinced that blunt dissection is more harmful to the nerves. This is also in contrast to the study of Kelly et al. (2006), as the muscle split in their patients with persisting electromyographical irregularities were all performed by sharp dissection. According to our results, the nerves supplying the distal part of the vastus medialis muscle—especially the transverse coursing branches and the intramuscular anastomoses—are prone to be severed using the midvastus approach. However, further clinical studies are necessary to clarify whether such a possible denervation may lead to altered patellar tracking, which may result in long-term functional problems.

In summary, the subvastus approach seems to offer the best possibility for a nerve-sparing way into the knee joint. Performing the medial parapatellar approach, no intramuscular nerve branches are harmed; only the sensory branches terminating within the ventromedial part of the capsule of the knee joint will likely be disrupted. Finally, using the midvastus approach, one cannot exclude the possibility of severing intramuscular nerve branches. As the approach performed depends on several factors, our results will provide additional valuable decision support.  
